# Biocompatible β-cyclodextrin-based metal-organic frameworks

**DOI:** 10.3389/fchem.2025.1682298

**Published:** 2025-12-01

**Authors:** Kirstin Wilson, David B. Cordes, Aidan P. McKay, Aaron B. Naden, Oxana V. Magdysyuk, Daniel N. Rainer, A. Robert Armstrong, Russell E. Morris, Aamod V. Desai, Romy Ettlinger

**Affiliations:** 1 EaStCHEM School of Chemistry, University of St Andrews, St Andrews, United Kingdom; 2 School of Chemistry and Chemical Engineering, University of Southampton, Southampton, United Kingdom; 3 Department of Chemistry, Indian Institute of Technology Madras, Chennai, India; 4 TUM School of Natural Sciences, Department of Chemistry, Technical University of Munich, Munich, Germany

**Keywords:** cyclodextrin, metal-organic frameworks, biocompatible MOFs, crystal growth, single crystal X-ray diffraction

## Abstract

β-Cyclodextrin (β-CD) is a cyclic heptasaccharide, part of the family of molecules which are widely used in several biological applications. The unique cone-shape of cyclodextrins with multiple binding sites make them a well-suited building block for constructing porous crystalline solids, such as metal-organic frameworks (MOFs). However, owing to the symmetry constraints, progress in the coordination chemistry of β-CD with alkali and alkaline earth metal cations has been limited and there is less understanding of this chemistry compared to its analogues of α-CD and γ-CD. In this work, synthetic conditions were optimised to obtain two MOF structures with β-CD as the organic linker, one each with Na^+^ and K^+^ cations. As well as structural determination, detailed solid-state characterization is reported for both the MOFs. The structure analysis helps shed light on the binding tendencies of β-CD and these structures will further facilitate the deployment of biocompatible building blocks for the development of reticular solid materials.

## 1 Introduction

In a world of increasing pollution, more attention needs to be drawn to the preparation of naturally biocompatible materials. The rich, reticular chemistry of the inorganic-organic hybrid material class of metal-organic frameworks (MOFs) allows the creation of novel materials following the EU Commission Recommendation of ‘safe and sustainable by design’ (European Comission, 2022). This can be achieved by the careful selection of inorganic and organic building blocks that degrade safely and effectively in biological systems, making them suitable for various biomedical and environmental applications. In case of inorganic building blocks, the chosen metal ideally should be categorised as an essential metal for humans: they must be present in human tissue, their absence in the human body would cause irreversible and severe damage, and their supplementation can normalise the reduction of physiological functions. Overall, three groups of such essential metals can be distinguished: *i)* bulk biological metals (Na^+^, K^+^, Mg^2+^, Ca^2+^, Fe^2+^, and Fe^3+^), *ii)* essential trace elements (Co^2+^, Cu^2+^, and Zn^2+^), and *iii)* possibly essential trace metals (Ni^2+^) (Zoroddu et al., 2019). In the case of the organic building blocks, different types of ligands are considered as biocompatible, including amino acids, peptides, nucleobases, saccharides, porphyrins, and proteins. Combining these building units, will ensure the resulting material environmentally is friendly and potentially useful in applications such as drug delivery and food packaging where more traditional MOFs have previously faced limitations (Li et al., 2017; Rajkumar et al., 2019).

**SCHEME 1:**
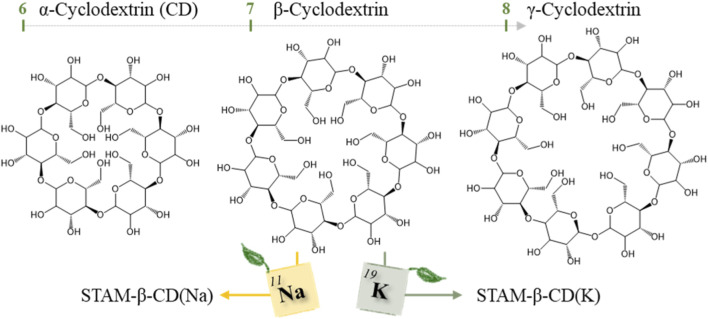
Overview of α, β and γ-Cyclodextrin (CD) and the use of β-CD for preparing STAM-β-CD(Na) and STAM-β-CD(K) MOFs.

Within the class of saccharides, cyclodextrins (CDs) have attracted more and more attention: they are natural and biodegradable cyclic oligosaccharides, that are composed of 6, 7, or 8 D-glucopyranose units linked with α-1,4-glycosidic linkages, corresponding to α, β and γ-CD, respectively (Scheme 1) (Dummert et al., 2022; Rajkumar et al., 2019; Roy and Stoddart, 2021). They provide many important properties including being non-toxic, non-carcinogenic, water soluble and biocompatible. This allows their utilization in a variety of industries such as cosmetics, food and pharmaceuticals (Dummert et al., 2022; Roy and Stoddart, 2021; Xu et al., 2023). These CD structures adopt cone or bucket like structures, in the case of the β-CD featuring a depth of approximately 0.8 nm and an inner diameter of 0.78 nm (Dummert et al., 2022; Rajkumar et al., 2019; Roy and Stoddart, 2021). While their hydrophobic core facilitates the binding of hydrophobic guest molecules and yields the resulting stability and solubility properties, their hydrophilic exterior consists of hydroxyl groups which provide water solubility (Blight et al., 2020; Dummert et al., 2022; Hartlieb et al., 2017). These hydroxyl groups accommodate a plethora of functional groups, and along with ether groups these are electron rich and contain lone pairs enabling easy coordination to metals to form CD-based metal-organic frameworks (MOFs) (Kang et al., 2021).

To date, most of the reported CD-MOFs only feature alkali metals (Na ^+^, K^+^), as their respective hydroxides can be used as deprotonating agents–thereby determining the number of possible achievable structures (Jia et al., 2019; Li et al., 2020; Li et al., 2021; Nadar et al., 2019; Sha et al., 2016; Volkova et al., 2020). In case of γ-CD, another approach is the introduction of a transition metal, such as Cu^2+^, to increase the M-O bond strength and the overall stability of the MOF (Xu et al., 2016). The well-known CD-MOF-1 has previously been crystallised as cubic crystals in the *I*432 space group using the 8-unit γ-CD (Roy and Stoddart, 2021). MOFs based on β-CD often yield more irregular shapes and morphologies (Dummert et al., 2022; Xu et al., 2023), are harder to crystallise and/or often display poor crystallinity due to the C7 rotational symmetry of the 7-unit β-CD structure (Blight et al., 2020).

In this work, we focused on optimizing the synthesis conditions to prepare edible, biocompatible and non-toxic MOFs based on β-CD using green and inexpensive processes, STAM-β-CD(M) (where STAM stands for St Andrews MOF, and M stands for the alkali metal used, either Na or K). The resulting materials were structurally investigated with single crystal X-ray diffraction (SC-XRD), and the bulk samples were characterised using several techniques, including, variable temperature powder X-ray diffraction (VT-PXRD), Fourier-transformed infrared (FT-IR) spectroscopy, thermogravimetric analysis (TGA) and scanning electron microscopy (SEM).

## 2 Materials and methods

### 2.1 Materials

The reagents, sodium hydroxide (NaOH; Fisher Scientific), potassium hydroxide (KOH; Fisher Scientific), β-cyclodextrin (β-CD; Sigma Aldrich), hexadecyltrimethylammonium bromide (CTAB; Sigma Aldrich), methanol (MeOH; Fisher Scientific), and ethanol (EtOH; Fisher Scientific), were obtained commercially and used as received.

### 2.2 Synthesis

The synthesis of STAM-β-CD(Na) and STAM-β-CD(K) MOFs was performed using three different approaches, either via slow diffusion (2.2.1 Method-1), solvothermal (2.2.2 Method-2), or surfactant-assisted (2.2.3 Method-3).

#### 2.2.1 Method-1: Vapour diffusion

*STAM-β-CD(Na)*: β-CD (0.5675 g, 0.5 mmol) and sodium hydroxide (0.40 g, 10 mmol) were dissolved in water (10 mL). 5mL of this solution was added to a 7 mL borosilicate sample vial. The sample vial was added to a vial containing methanol (15 mL). This was left at room temperature where vapour diffusion occurred, and the methanol evaporating from the large vial and recondensing in the sample vial. After 14 days there had been no precipitation, so the experiment was repeated but the solution left at 30 °C for 3 days and then 60 °C for a further 4 days. Again, no precipitation occurred so cetyltrimethylammonium bromide (CTAB) (8 mg per 1 mL stock, 2.2 × 10^−5^ mol) was added to both experiments and the solutions left at 50 °C. After a further 12 days both solutions had formed crystals.

*STAM-β-CD(K)*: β-CD (0.5675 g, 0.5 mmol) and potassium hydroxide (0.5610 g, 10 mmol) were dissolved in water (10 mL). 5mL of this solution was added to a 7 mL borosilicate sample vial. The sample vial was added to a vial containing methanol (15 mL). This was left at room temperature where vapour diffusion occurred (Blight et al., 2020). Crystals grew as the concentration of methanol in the solution increased.

#### 2.2.2 Method-2: Solvothermal

*STAM-β-CD(Na) and STAM-β-CD(K)*: β-CD (2.4 g, 2.1 mmol) and sodium hydroxide (0.71 g, 18 mmol) or potassium hydroxide (1 g, 18 mmol) were added to a mixture of 10 mL water and 20 mL ethanol (ratio 4:6, 30 mL in total) solution. This was stirred at room temperature for 30 min and then heated in an oven at 100 °C for 3 days. This resulted in crystals forming in solution but also the formation of an unidentified gel. The crystals were filtered and washed with ethanol.

#### 2.2.3 Method-3: Surfactant-assisted crystallisation using CTAB

*STAM-β-CD(Na) and STAM-β-CD(K)*: β-CD (0.28375 g, 0.25 mmol) and sodium hydroxide (0.2 g, 5 mmol) or potassium hydroxide (0.28 g, 5 mmol) were dissolved in water (5 mL). The solution was added to a sample vial in a flask containing methanol. This was heated at 30 °C overnight to allow the methanol to diffuse into the sample vial. Subsequently, this stock solution (7 mL) was then added to a sample vial with CTAB (56 mg, 0.15 mmol) and left at 50 °C for 4 days. The crystals formed were filtered and washed with ethanol. The method is based on a literature crystallisation of γ -CD-MOFs (Furukawa et al., 2012).

### 2.3 Structural characterization

Powder X-ray diffraction (PXRD) patterns were recorded using Mo K_α1_ radiation (λ = 0.70930 Å) on STOE STADIP diffractometer at room temperature from 1.5° to 27.5° (2θ) in capillary Debye–Scherrer mode. For variable temperature PXRD (VT-PXRD), samples were measured by filling a quartz capillary (0.7 mm) and were gradually heated and cooled from 20 °C to 220 °C, in intervals of 20 °C using a Cryostream with N_2_ gas flow. The temperature ramping rate was 10 °C/min with 2 min waiting time before each measurement started, the measurements were carried out in fixed detector mode within a 2θ range of 1.5°–20.3°. TGA and differential thermal analysis (DTA) were recorded under nitrogen gas on a Stanton Redcroft STA-780 from room temperature to 700 °C, with a heating rate of 5 °C/min under N_2_ gas flow. Scanning electron microscopy (SEM) images were obtained by placing the dried powder of the samples over an aluminium tape and coating it with gold using a Quorum Q150R ES coater at 20 mA current for 30 s. All FT-IR spectra were obtained on a Shimadzu IR Affinity-1S IR Spectrometer with an ATR attachment in the range of 4000 cm^−1^ to 400 cm^−1^.

Single-crystal X-ray diffraction data for compounds STAM-β-CD(Na) and STAM-β-CD(K) were collected at 125 or 141 K using a Rigaku MM-007HF High Brilliance RA generator/confocal optics [Cu Kα radiation (λ = 1.54187 Å)] with XtaLAB P200 diffractometer. Intensity data for all compounds analysed were collected using either CrystalClear (Rigaku Corporation, 2015) (using ω steps and accumulating area detector images spanning at least a hemisphere of reciprocal space) or CrysAlisPro (Rigaku Oxford Diffraction, 2023) (using a calculated strategy), and processed (including correction for Lorentz, polarization and absorption) using CrysAlisPro. Structures were solved by dual space methods (SHELXT (Sheldrick, 2015a)) and refined by full-matrix least-squares against F^2^ (SHELXL-2019/3 (Sheldrick, 2015b)). Non-hydrogen atoms were refined anisotropically, and carbon-bound hydrogen atoms were refined using a riding model. Hydroxyl hydrogen atoms were placed in calculated positions, rotated into appropriate hydrogen bonding positions where possible and refined using a riding model. Sodium- and potassium-coordinated oxygen atoms were treated as aqua ligands with hydrogen atoms placed in idealised positions by Olex2 and rotated into chemically reasonable positions, prioritising hydrogen bonding where possible, and refined using a riding model. Several parts of the cyclodextrin molecules in STAM-β-CD(Na) showed positional disorder. This was refined over two sites in each case, with restraints to bond distances and thermal motion. In STAM-β-CD(K) two potassium sites (K8 and K9) showed excessive thermal motion when refined at full occupancy. Their occupancies were refined, showed convergence at close to 50% occupancy each, and then were fixed at half occupancy. An oxygen bridging K5 and K7 (O288) showed disorder over two positions as well as a third disordered position coordinated only to K7. The occupancy of these three oxygens was refined with all three sites summed to one and with restraints on K-O distances. Two other coordinated oxygens also showed disorder over two sites. Both structures showed high proportions of void space (STAM-β-CD(Na): 3226 Å^3^, STAM-β-CD(K): 1894 Å^3^) and the SQUEEZE (Spek, 2015) routine implemented in PLATON (Spek, 2009) was used to remove the contribution to the diffraction pattern of the unordered electron density in the void spaces. After refinement following SQUEEZE in STAM-β-CD(Na), five sites in close proximity to hydrogen-bonding groups were identified with sufficient residual electron density to require modelling as atomic sites. These sites (O288-O292) were modelled as complete (O288) or partially-occupied oxygen sites and refined isotropically. These oxygens are probably fragments resulting from hydrogen-bonded solvent, where the only (partially) ordered part of the solvent is the oxygen site. All calculations except SQUEEZE were performed using the Olex2 interface. (Dolomanov et al., 2009). Selected crystallographic data are presented in Supplementary Table S1. CCDC 2456464-2456465 contains the supplementary crystallographic data for this paper. These data can be obtained free of charge from The Cambridge Crystallographic Data Centre via www.ccdc.cam.ac.uk/structures.

## 3 Results and discussion

### 3.1 Synthesis and structural features

The synthesis of the two MOFs was attempted using three different routes. The slow vapour diffusion method has been the most successful method for preparing several cyclodextrin MOFs (Dummert et al., 2022). However, in the current case, precipitation or crystallisation was not observed, even when the solutions were left standing for up to 2 weeks. Solvothermal synthesis has been routinely employed for the preparation of MOFs with non-cyclodextrin ligands, and a conventional solvothermal approach was tested as well. Although crystals were obtained in the solution upon cooling, a gelatinous precipitate formed alongside, making isolation of the pure CD-MOF difficult. Surfactant-assisted crystallisation has found increasing success for preparing a range of MOFs, and since its advent the controlled crystal growth of several well-characterised MOFs has been achieved (Ma et al., 2011), including for γ-CD-MOFs and K-β-CD-MOF (Furukawa et al., 2012; Sadeh et al., 2023). In the current work, cetyltrimethylammonium bromide (CTAB) was employed as the nucleating agent, and crystals of the pure product were obtained for both STAM-β-CD-MOFs. The crystals were found suitable for single-crystal X-ray diffraction (SC-XRD) studies, and the structures of the two MOFs were determined and the bulk phase of the materials characterised further.

**FIGURE 1:**
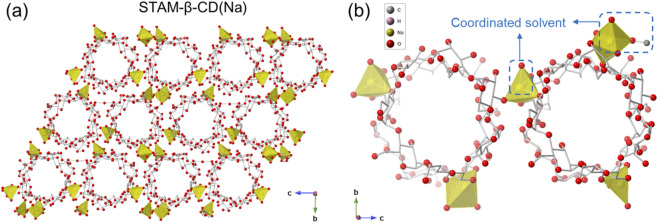
**(a)** Packing diagram of STAM-β-CD(Na) along *a*-axis, and **(b)** Local coordination environment around Na^+^ cation, showing coordinated solvent (water and methanol) molecules. (Colour code: Na, yellow; O, red; C, grey. H-atoms and uncoordinated solvent molecules are omitted for clarity. Na atoms represented as polyhedral; O atoms are represented in balls; C atoms are shown as sticks).

STAM-β-CD(Na) crystallised as colourless prism-shaped crystals in space group *P*2_1_ (Supplementary Table S1). The asymmetric unit consists of four units of β-CD and five Na^+^ cations (Supplementary Figure S1). The Na^+^ ions are coordinated to the secondary alcoholic groups of the ligand, as well as to both solvents used in the synthesis, water and methanol (Figure 1b, SI 1, SI 2). The structural packing leads to formation of 1-dimensional nanotubular channels (Figure 1a). The channel walls are formed by linkage of adjacent β-CD units held by Na^+^ cations at triangular positions for each β-CD units, and all the Na^+^ cations are in the eclipsed positions along the nanoporous channels (Figure 1a, SI 3). The overall packing consists of corrugated sheets that are held together by non-covalent hydrogen bonding interactions between the primary alcohols of the adjacent layers (Supplementary Figure S4,5). The coordination bond distances (Na-O bonds) are in the range from 2.266 (6) to 2.845 (9) Å. The porous channels are parallel to each other, and the pore windows are approximately rectangular with dimensions between ca. 7 to 8 Å (Supplementary Figure S6). It is worth highlighting, that formation of Na-MOFs with β-CD is rare (Supplementary Table S2), with the only other known structure significantly different to STAM-β-CD(Na) (Lu et al., 2015; Sha et al., 2015). The differences in synthesis conditions may partly be responsible for the observed differences in structures.

**FIGURE 2:**
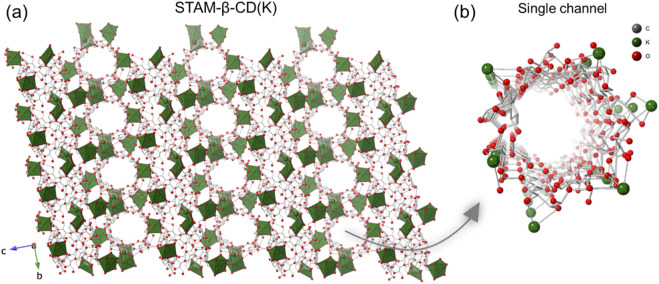
**(a)** Packing diagram of STAM-β-CD(K) along *a*-axis, and **(b)** perspective view of a single nanoporous channel in the MOF. (Colour code: K, green; O, red; C, grey. H-atoms and solvent molecules occupying the voids are omitted for clarity. K atoms represented as polyhedral in **(a)** and balls in **(b)**; O atoms are represented in balls; C atoms are shown as sticks).

STAM-β-CD(K) crystallised in the space group *P*1, with the asymmetric unit consisting of four cyclodextrin molecules and eight K^+^ ions (nine sites in total, two with half occupancy) (Supplementary Table S1; Supplementary Figure S7). The structure also contains both water and methanol solvent molecules, which are found between adjacent cyclodextrin molecules. The cyclodextrin rings are arranged in two nanotubular groups, nearly perpendicular to each other, and both nanoporous channels are linked by K^+^ ions leading to formation of a 3D-network (Figures 2a, Supplementary Figure S8, 9). The K-O bond distances range from 2.667 (10) to 3.137 (11) Å and includes the coordination of O atoms of the primary alcohols and ring O atoms of β-CD, as well as of coordinated water. It is also worth noting that the shape and size of the pore channels in STAM-β-CD(K) are considerably different compared to that in STAM-β-CD(Na), with a more cylindrical shaped pore channel and pore window dimensions of ca. 6.3-6.6 Å (Supplementary Figure S10). STAM-β-CD(K) features several hydrogen-bonding interactions, both intramolecular (between same β-CD unit) and intermolecular (between neighbouring β-CD moieties) (Supplementary Figure S11). In addition to the K^+^ ion coordination that holds the perpendicular arrangement of nanotubes together, hydrogen-bonding interactions with the uncoordinated solvent molecules (methanol) support the structure (Supplementary Figure S12). Compared to other known structures of K-β-CD-MOFs (Supplementary Table S3), the structure in the current work is closely related to one previously reported compound (β-CDMOF-1) (Blight et al., 2020), both showing similar unit cells, as well as similar to crystal morphology. Interestingly, both MOFs were prepared by different methods: STAM-β-CD(K) using a nucleating agent and gentle heating, whereas β-CDMOF-1 using slow diffusion having methanol and water as the two solvents.

### 3.2 Advanced material characterization

**FIGURE 3:**
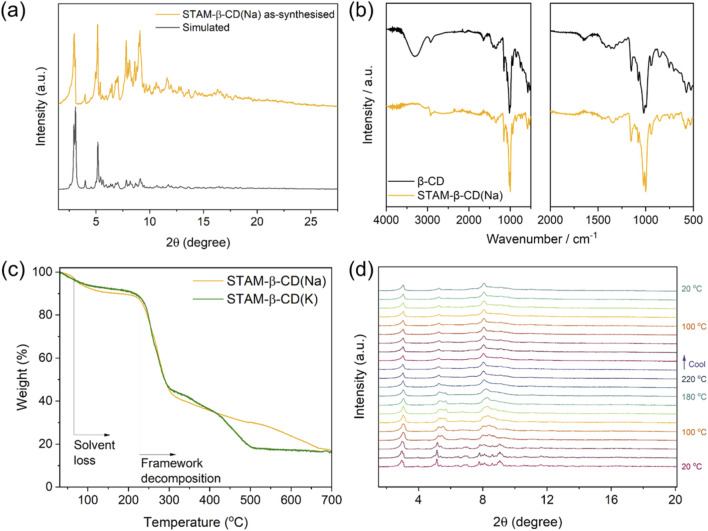
**(a)** PXRD profiles for STAM-β-CD(Na), simulated (grey) and as-synthesised phase (dark yellow). **(b)** FT-IR spectra for FT-IR spectra for β-CD (black) and as-synthesized STAM-β-CD(Na) (dark yellow). **(c)** TGA profiles for STAM-β-CD(Na) (yellow) and STAM-β-CD(K) (green). **(d)** VT-PXRD profiles for STAM-β-CD(Na) for heating the sample in a sealed capillary up to 220 °C, in stepwise increments of 20 °C.

Both of the MOFs were further characterised using several techniques. The IR spectra of both STAM-β-CD(Na) and STAM-β-CD(K) are similar to the spectrum associated with β-CD (Figure 3b, SI 15), but show a loss of the broad -OH vibrational band between 3600 and 3100 cm^-1^.

The PXRD pattern for STAM-β-CD(Na) validated the purity of the bulk phase, with a close agreement of the peaks with the calculated profile (Figure 3a). In the case of STAM-β-CD(K), the bulk phase had a slightly different pattern from the calculated profile (Supplementary Figure S13), suggesting structural changes upon removal of the sample from its mother liquor. Such a behaviour has been previously noted for a similar MOF, (Blight et al., 2020), and attributed to the loss of solvent molecules. Such soft, flexible structures are well documented in the literature of MOFs. (Schneemann et al., 2014). In the case of STAM-β-CD(K), a subtle structural change is not unexpected, especially as the solvent molecule plays a key role in the assembly of the framework (Supplementary Figure S12). SEM images revealed considerable differences between the two MOFs (Supplementary Figure S16). STAM-β-CD(Na) had aggregation of irregular shaped particles, whereas STAM-β-CD(K) exhibited plate like morphology and had stacked aggregates.

The thermal properties of the MOFs were initially evaluated by measuring the TGA profile. Both the MOFs showed a loss of solvent molecules below 100 °C, and a stable mass profile up to ca. 220 °C (Figure 3c). After this a sharp mass loss followed by further stepwise loss, which could be attributed to the decomposition of the structure. Commercially available β-CD is reported to have thermal stability up to 250 °C–400 °C (Trotta et al., 2000), which is reduced by the formation of the MOFs. It is worth noting, that a similar thermal profile has been reported for other CD-MOFs, including β-CDMOF-1 (Blight et al., 2020). The formation of high porosity and low crystal density might be responsible in reducing the extent of non-covalent interactions in β-CD, causing slightly decreased thermal stability following MOF formation. To understand the temperature dependence of structural changes, the samples were heated incrementally up to 220 °C, and cooled stepwise to 20 °C in a sealed capillary tube and PXRD patterns were recorded at each temperature point. For both the MOFs, the crystallinity was retained throughout the heating and cooling cycles (Figure 3d, Supplementary Figure S15). In the case of STAM-β-CD(Na), a gradual shift in the positions of high intensity peaks and some disappearances of minor peaks are noticed up to 100 °C (Figure 3d), corresponding to the loss of solvent molecules (Figure 3c). Thereafter, heating up to 220 °C causes minimal structural changes. The crystalline phase formed at 220 °C remains unaltered in the cooling cycle, with no change in peak positions or appearance of new peaks. For STAM-β-CD(K), the major structural changes occur only above 180 °C, where broadening of peaks is observed with the peak at ca. 3° 2θ becoming the sharpest peak (Supplementary Figure S16). Similar to the observation for STAM-β-CD(Na), the phase formed for STAM-β-CD(K) upon heating undergoes no change in the crystallinity in the cooling cycle (Supplementary Figure S14). Elucidating the structures of the phases after the heat-treatment proved unachievable. No single-crystals amenable to analysis by SC-XRD were found. Similarly, tackling the issue using 3D ED revealed only very weakly diffracting particles, insufficient for crystal structure solution. In a separate experiment, samples of both the MOFs were heated up to 200 °C, and their SEM images were recorded upon cooling (Supplementary Figure S15). For the heated samples, the surface roughness is considerably higher along with smoother edges for the particles. Although the plate-like morphology was retained in the case of STAM-β-CD(K), the surface distortion was even more prominent.

## 4 Conclusion

In summary, the successful synthesis of two alkali cation MOFs with β-CD as the linker is reported. To overcome the symmetry constraints of the linker, several synthesis conditions were screened, and surfactant-assisted crystallisation was found to be the most effective and reproducible method. Crystals of both STAM-β-CD (Na) and STAM-β-CD(K) were obtained and their structures were determined using SCXRD. Both the MOFs have large porous channels, benefitting from the inherent characteristic of β-CD, and are rare examples of a porous, alkali cation MOFs. Detailed solid-state, bulk-phase characterisation was further carried out for the two MOFs, shedding light on their thermal stability. The synthesis strategies and structural features offer a new route to explore the chemistry of materials based on β-CD and facilitate the exploration of biocompatible building units in reticular solids.

## Data Availability

Complete experimental and refinement data are contained in the deposited CIFs along with structure factors and an embedded .res file, deposited in the CSD with CCDC reference codes CCDC 2456464-2456465. These data can be obtained free of charge from The Cambridge Crystallographic Data Centre via www.ccdc.cam.ac.uk/structures.
