# Supplementary material for: Biocompatible β-cyclodextrin-based metal-organic frameworks
Source: Front Chem. 2025 Dec 1;13:1682298. doi: 10.3389/fchem.2025.1682298 (PMC12703878; doi:10.3389/fchem.2025.1682298)
Supplement: Supplementary file 1 [file DataSheet1.docx]

Supplementary Material

Biocompatible β-Cyclodextrin-based Metal-organic Frameworks

Kirstin Wilson,^1^ David B. Cordes,^1^ Aidan P. McKay,^1^ Aaron B. Naden,^1^ Oxana V. Magdysyuk,^1^ Daniel N. Rainer,^2^ A. Robert Armstrong,^1^ Russell E. Morris,^1^ Aamod V. Desai,^1,3,^* and Romy Ettlinger^1,4,^*

^1^ EaStCHEM School of Chemistry, University of St Andrews, St Andrews, KY169ST, United Kingdom

^2^ School of Chemistry and Chemical Engineering, University of Southampton, Southampton, SO17 1BJ, United Kingdom

^3^ Department of Chemistry, Indian Institute of Technology Madras, Chennai 600036, India

^4^ TUM School of Natural Sciences, Department of Chemistry, Technical University of Munich, Lichtenbergstrasse 4, 85748 Garching, Germany

**Contents**

[1 Crystal Structure Details 2](#_Toc203903091)

[2 Infrared Spectroscopy 15](#_Toc203903092)

[3 Electron Microscopy 16](#_Toc203903093)

[4 References 17](#_Toc203903094)

# Crystal Structure Details


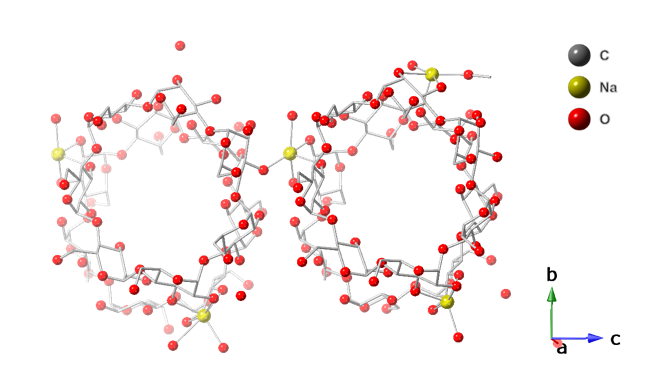


**Figure SI 1.** Asymmetric unit of STAM-β-CD(Na) (Colour code: Na, yellow; O, red; C, grey. H-atoms are omitted for clarity. O and Na atoms are shown in balls; C atoms as stick).


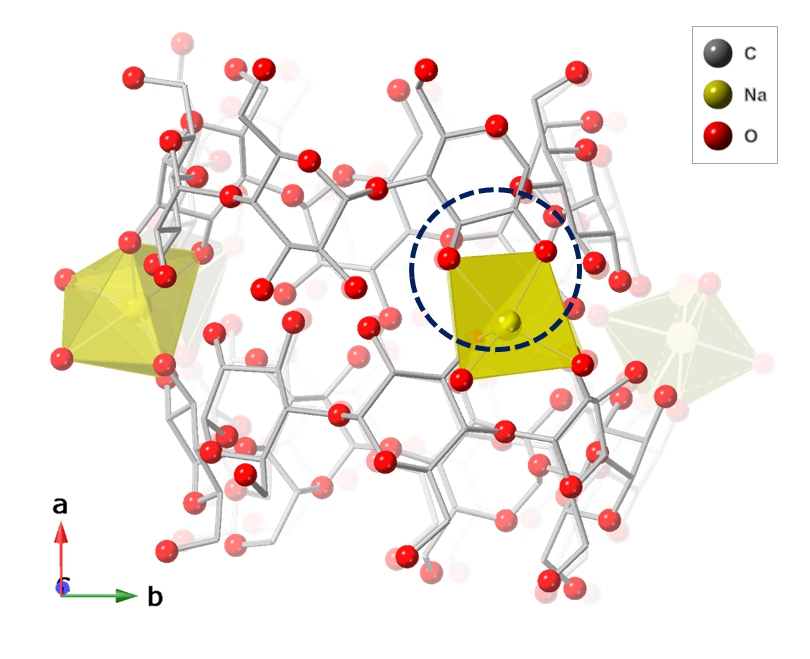


**Figure SI 2.** Single unit in STAM-β-CD(Na) showing coordination of secondary alcohols in the β-CD linkers with Na^+^ cations. (Colour code: Na, yellow; O, red; C, grey. H-atoms and uncoordinated solvent molecules occupying the voids are omitted for clarity. Na atoms are shown as polyhedral; C atoms as stick; O atoms as balls)

**
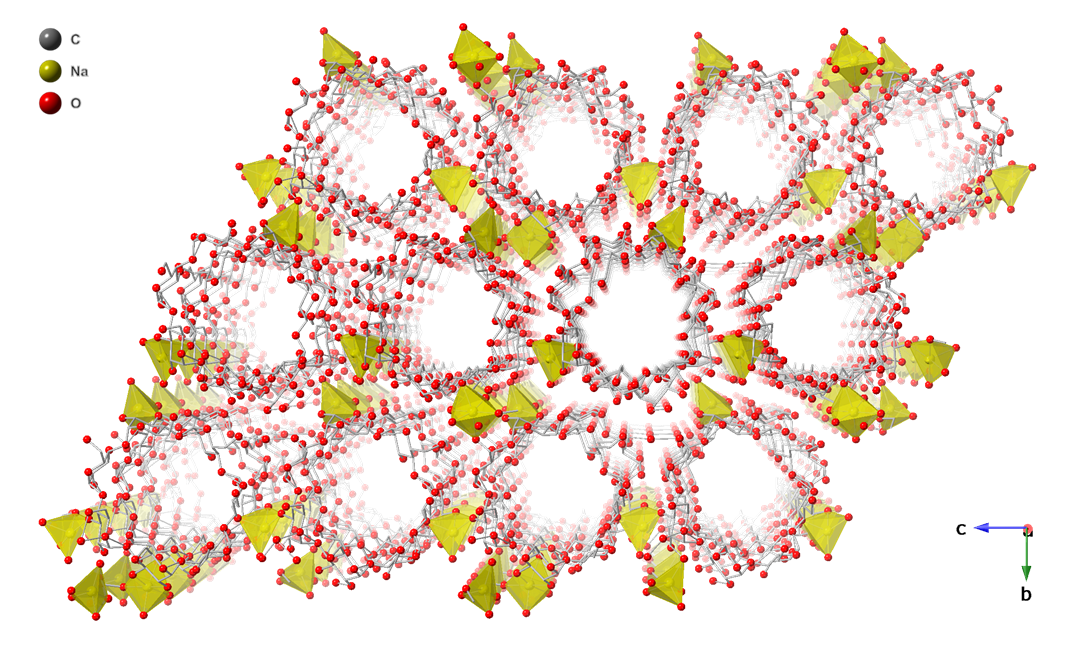
**

**Figure SI 3.** Perspective view of structural packing of Na- β-CD-MOF along the *a*-axis (Colour code: Na, yellow; O, red; C, grey. H-atoms and uncoordinated solvent molecules occupying the voids are omitted for clarity. Na atoms are shown as polyhedral; C atoms as stick; O atoms as balls).


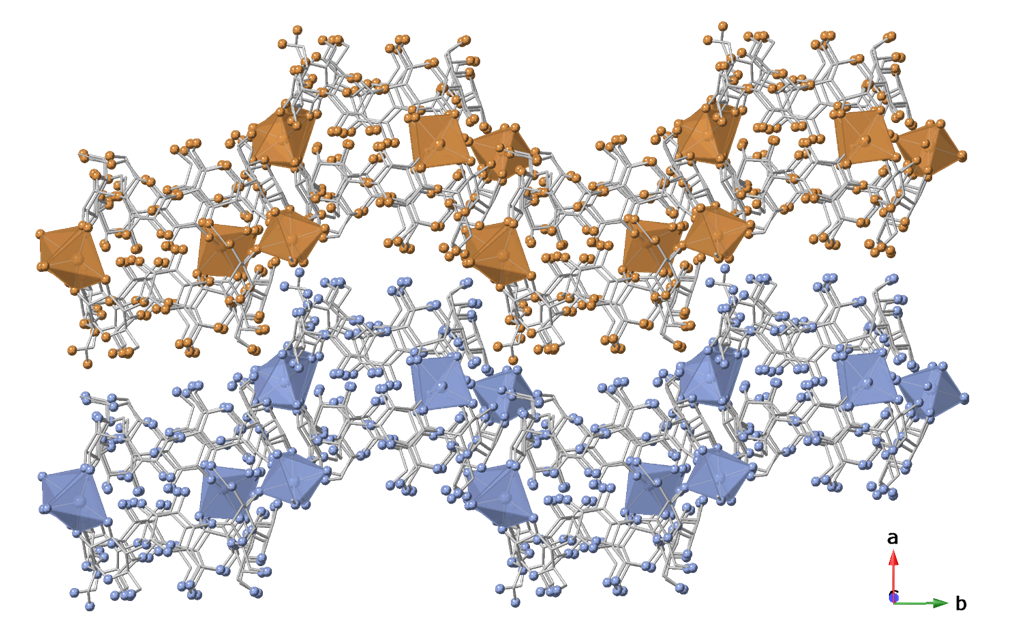


**Figure SI 4.** Figure showing two corrugated layers in STAM-β-CD(Na). (non-carbon atoms in each layer have the same colour; one brown and the other blue. Na atoms are shown as polyhedral; C atoms as grey sticks; O atoms as balls)


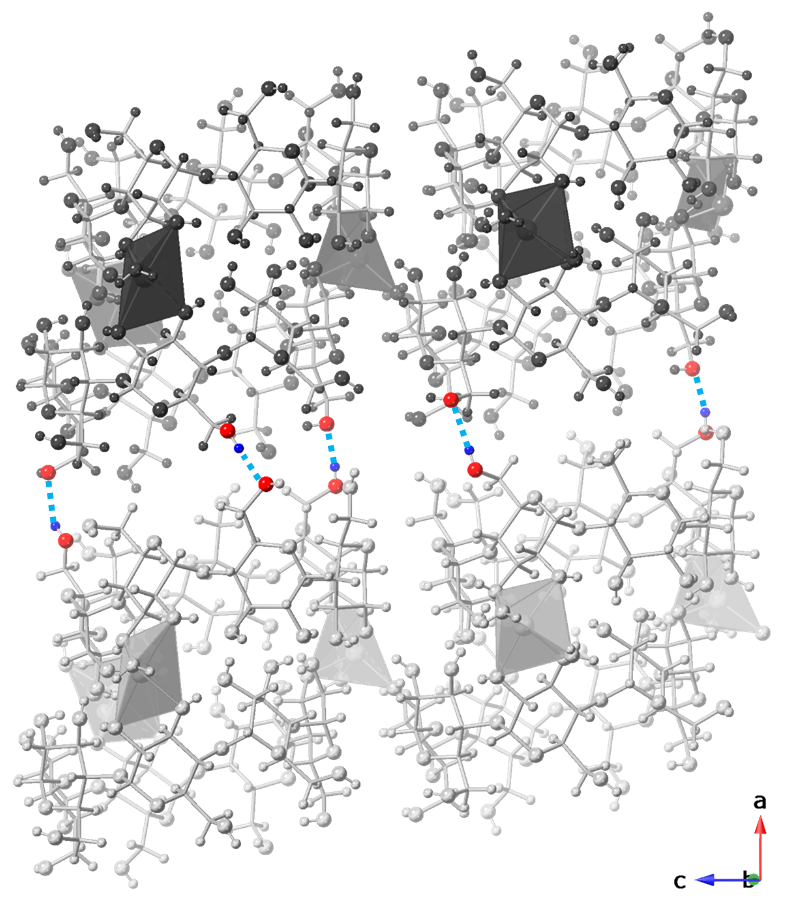


**Figure SI 5.** Figure showing the H-bonding interactions between primary alcohols in the adjacent 2D layers. (all the atoms in each layer have the same colour; one dark grey and the other light grey. Na atoms are shown as polyhedral; C atoms as stick; O atoms as balls. The atoms involved in H-bonds are coloured: O, red; H, blue; H-bond; sky blue dotted line).


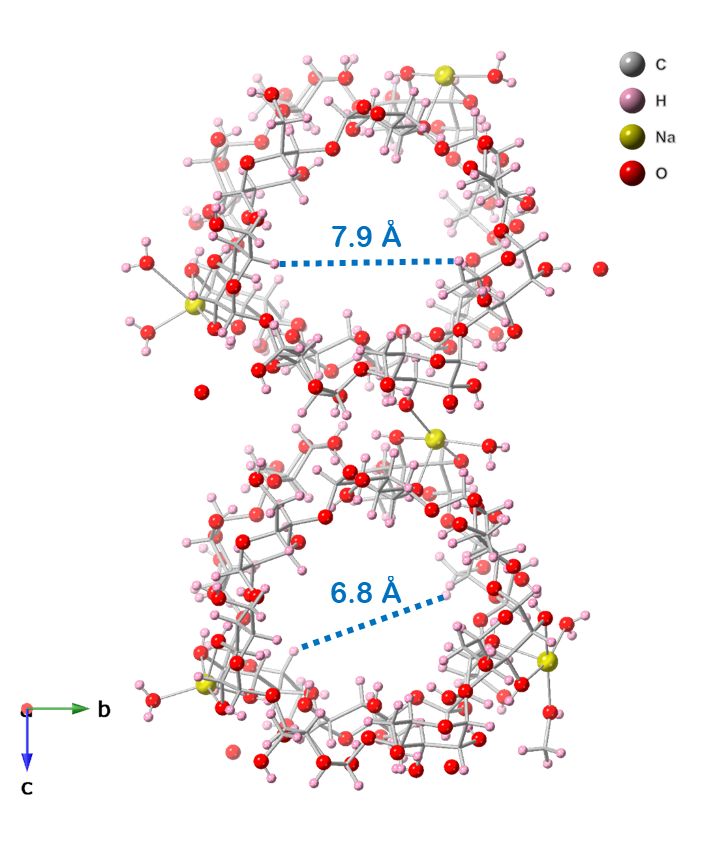


**Figure SI 6.** Figure showing the pore dimensions in the two channels in STAM-β-CD(Na). (Colour code: Na, yellow; O, red; C, grey; H, pink. C atoms as stick; Na, O and H atoms as balls. Distances are measured from H-atom to H-atom. Na, H and O atoms are shown as balls; C atoms as stick)


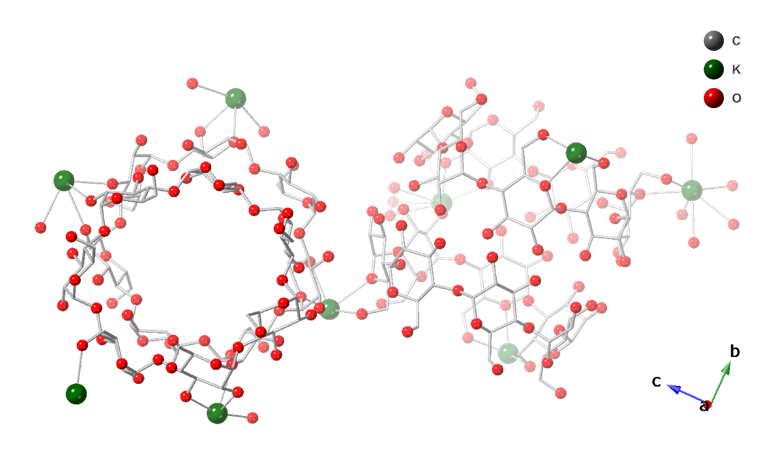


**Figure SI 7.** Asymmetric unit for STAM-β-CD(K). (Colour code: K, green; O, red; C, grey. H-atoms and uncoordinated solvent molecules occupying the voids are omitted for clarity. K and O atoms are shown as balls; C atoms as stick)

**
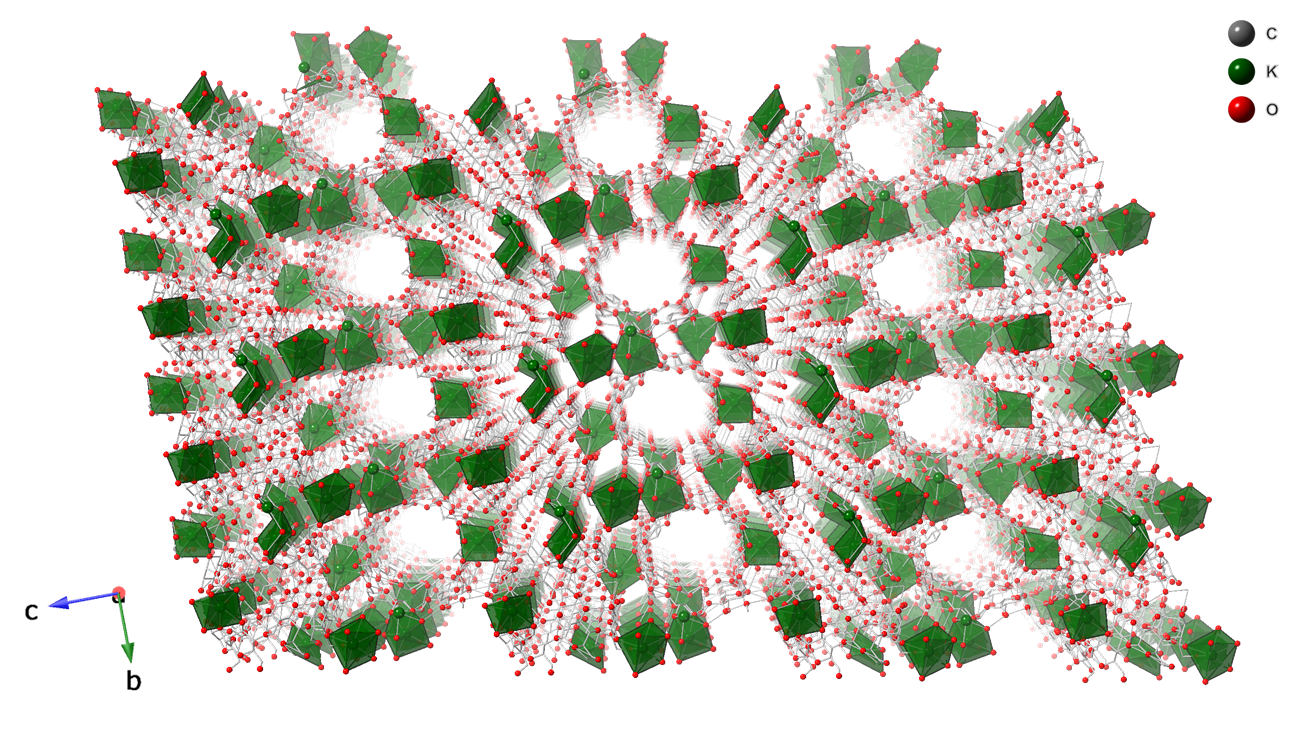
**

**Figure SI 8.** Perspective view of structural packing of STAM-β-CD(K) along the *a*-axis. (Colour code: K, green; O, red; C, grey. H-atoms and uncoordinated solvent molecules occupying the voids are omitted for clarity. K atoms are shown as polyhedral; C atoms as stick; O atoms as balls.)


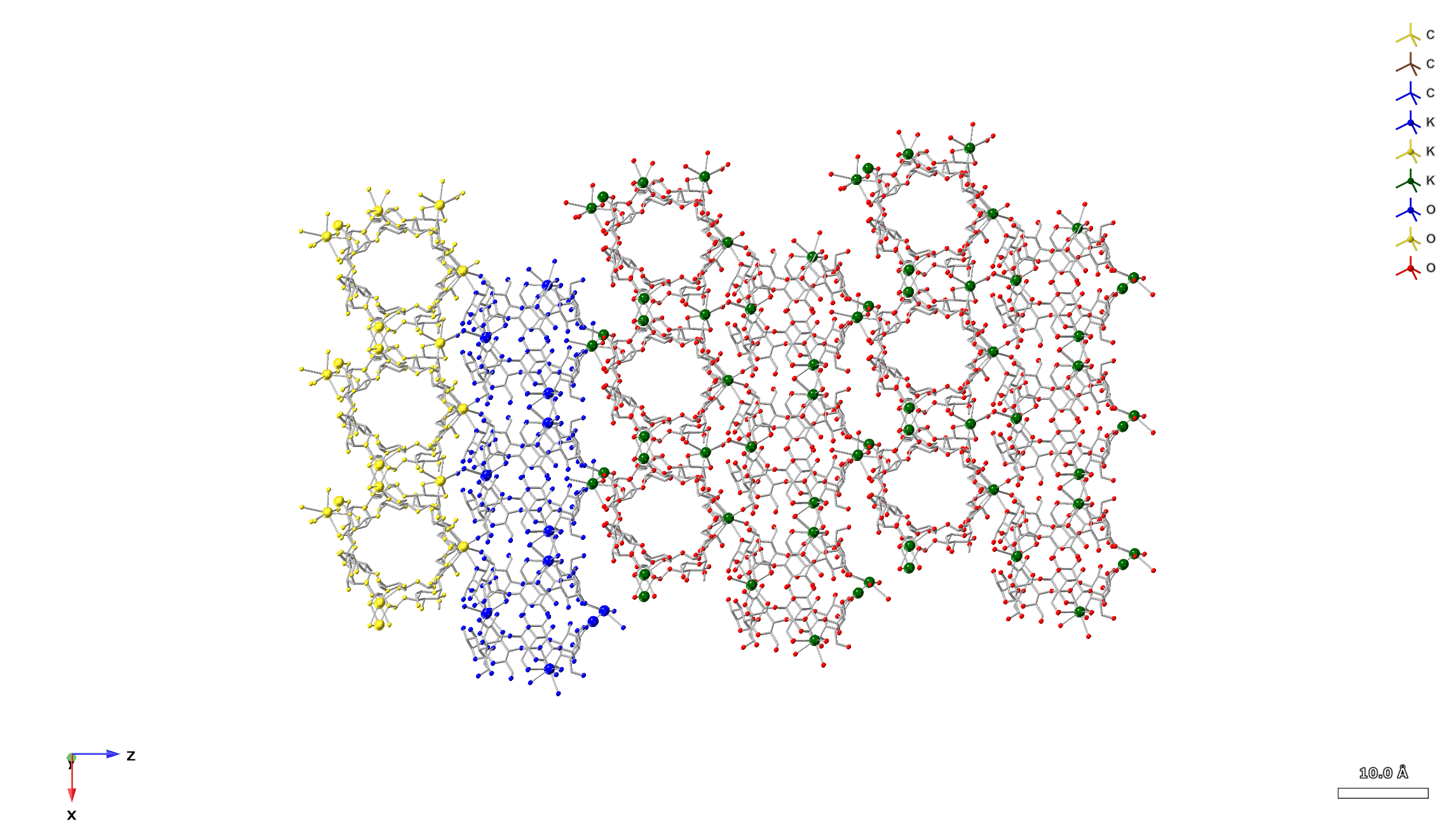


**Figure SI 9.** Packing diagram of STAM-β-CD(K). The atoms in the perpendicular nanotubular channels are highlighted and three channels are shown in yellow and one in blue on the left side of the figure. (K, green; O, red; C, grey. H-atoms and uncoordinated solvent molecules occupying the voids are omitted for clarity. K and O atoms are shown as balls; C atoms as stick)


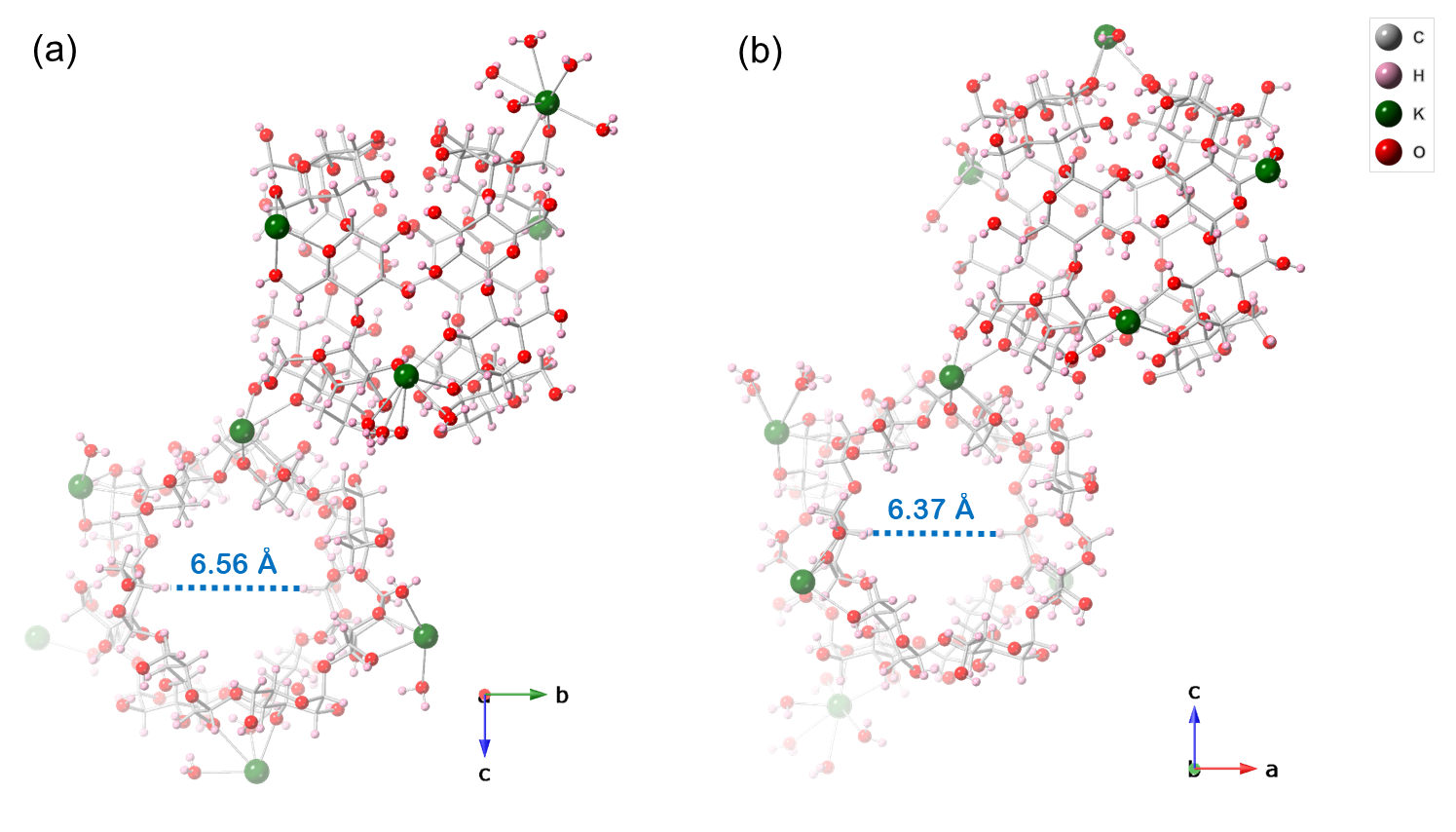


**Figure SI 10.** Figure showing the pore window dimensions measured between H-atoms along (a) *a*-axis and (b) *b*-axis. (Colour code: K, green; O, red; C, grey; H, pink. K, O and H atoms are shown as balls; C atoms as stick)


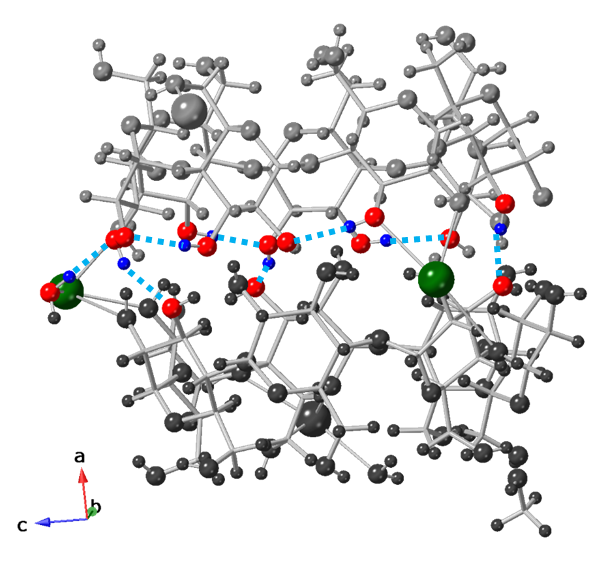


**Figure SI 11.** Diagram of two neighbouring units of β-CD in STAM-β-CD(K). The two shades of grey show different units, and the atoms highlighted showing a selection of those atoms involved in H-bonding interactions, either between the same unit or with the neighbouring moiety (colour code: All the atoms are shown in grey, except the ones highlighted. K, green; O, red; H, blue; H-bond, sky blue dotted line.


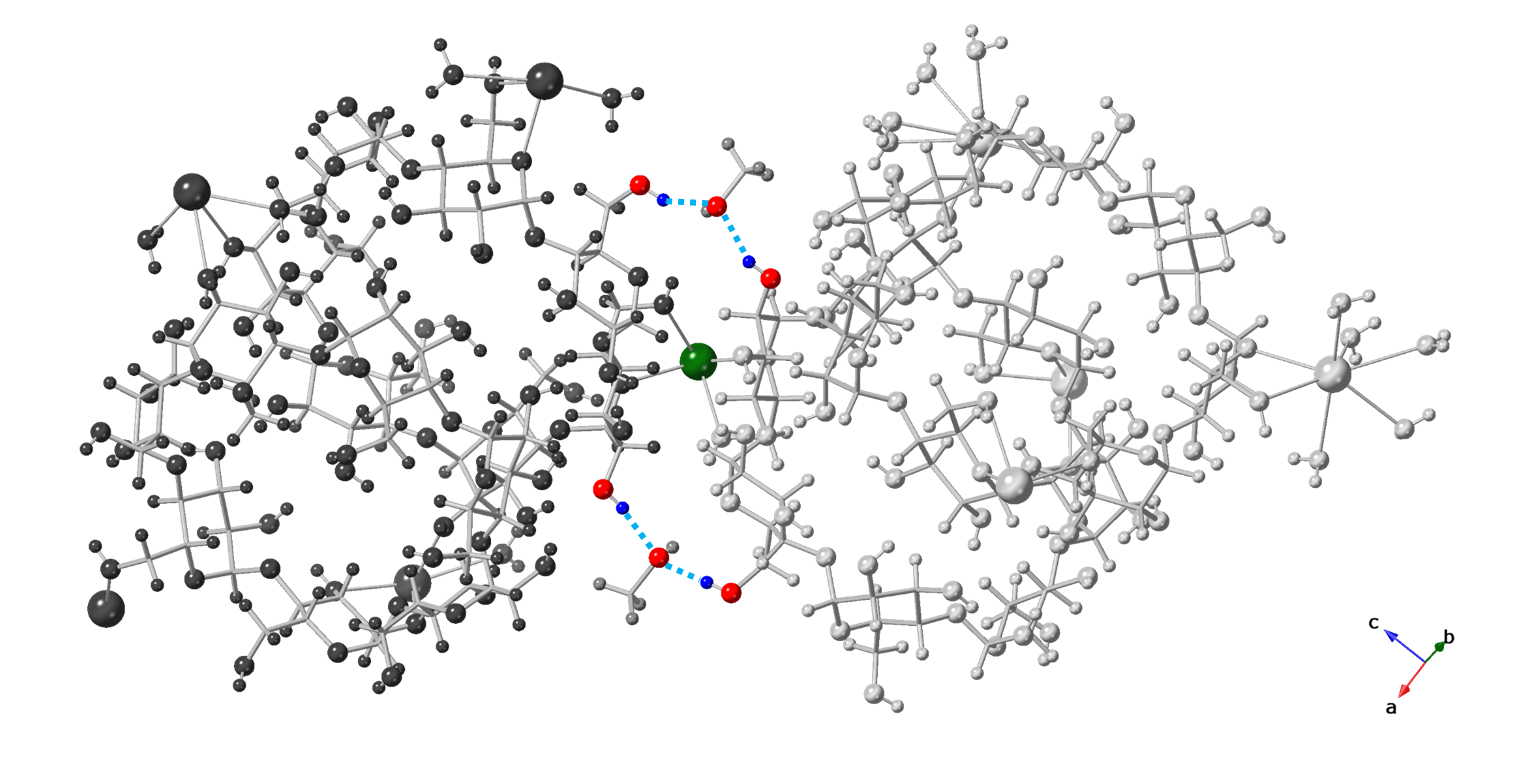


**Figure SI 12.** Diagram of STAM-β-CD(K) showing H-bonding interactions (orange dotted line) between the solvent molecule (methanol) and primary alcohols of the β-CD units of two different nanotubular channels (colour code: All the atoms are shown in grey, except the ones highlighted. K, green; O, red; H, blue; H-bond, dotted link in sky blue. The different shades of grey represent the two perpendicular sets of nanoporous channels and the highlighted K atom shows the one linking the two units).

**
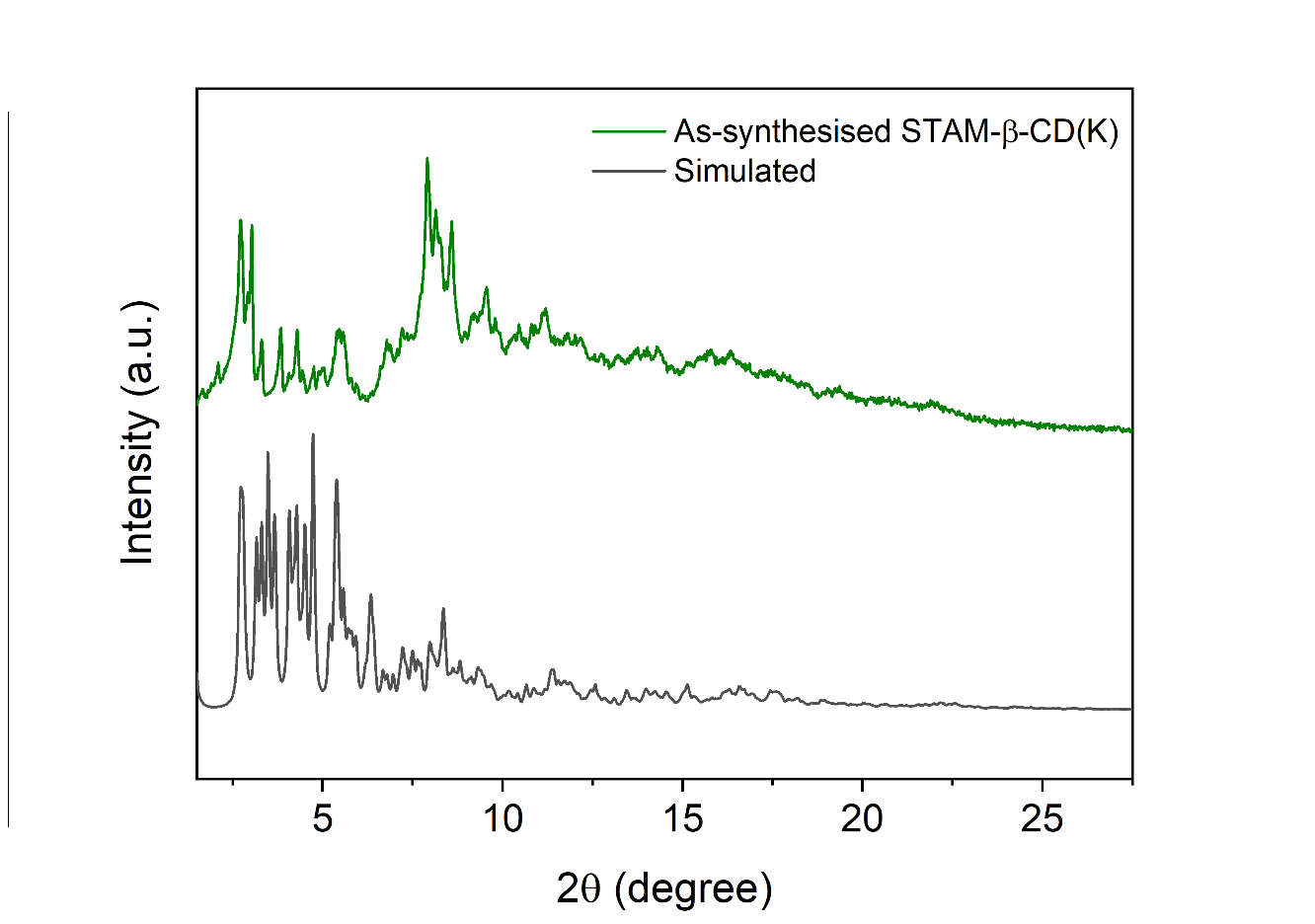
**

**Figure SI 13.** PXRD patterns for STAM-β-CD(K), simulated (grey) and as-synthesized phase (green).


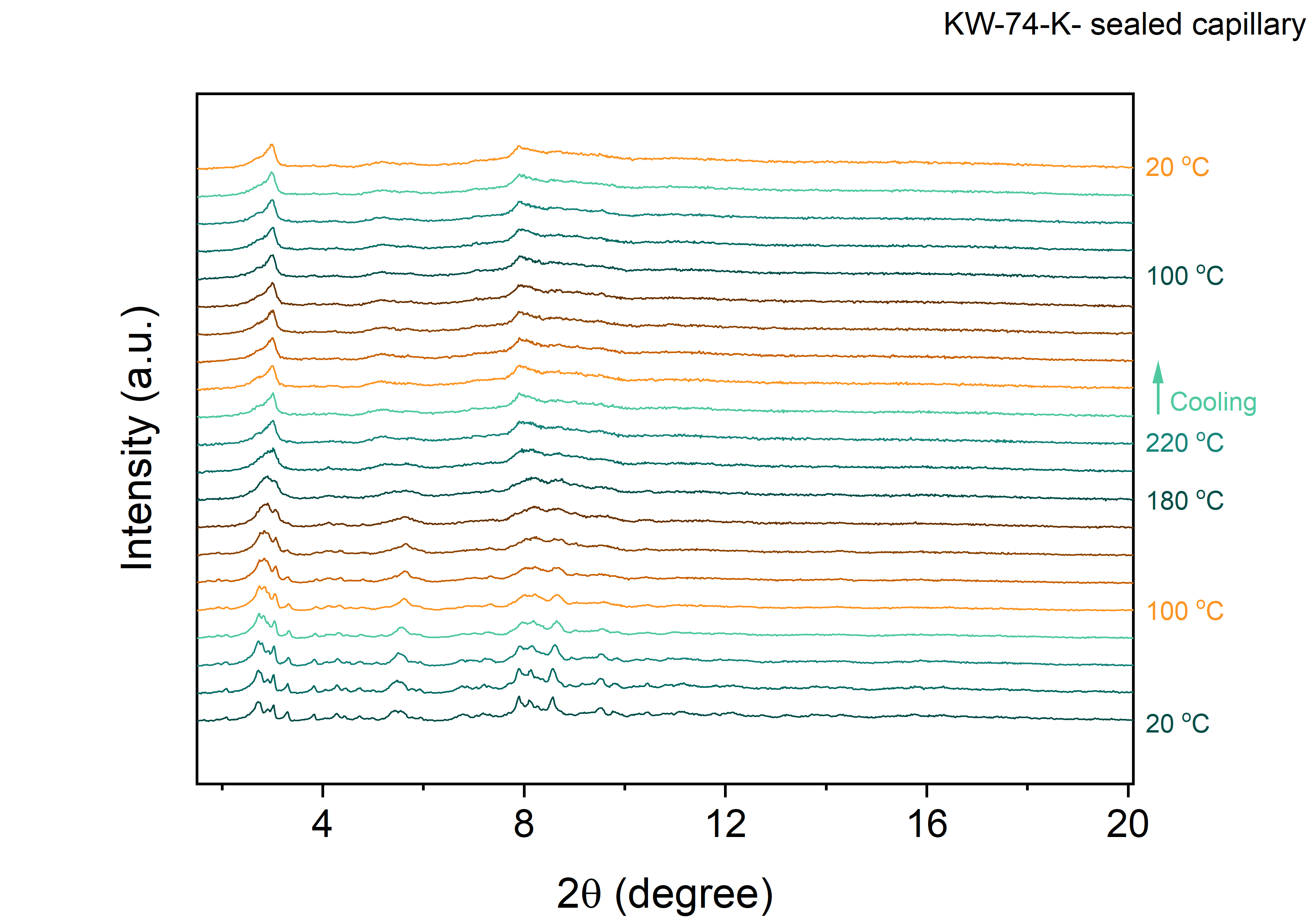


**Figure SI 14.** VT-PXRD profiles for STAM-β-CD(K) when heated in a sealed capillary up to 220 °C, in stepwise increments of 20 °C.

|  | **STAM-β-CD(Na)** | **STAM-β-CD(K)** |
| --- | --- | --- |
| formula | C_169_H_296_Na_5_O_150.25_ | C_170_H_306_K_8_O_151_ |
| fw | 4846.99 | 5078.93 |
| crystal description | Colourless prism | Colourless plate |
| crystal size [mm^3^] | 0.42×0.36×0.22 | 0.20×0.05×0.01 |
| temperature [K] | 125 | 141 |
| space group | *P*2_1_ | *P*1 |
| *a* [Å] | 15.91971(8) | 15.2321(5) |
| *b* [Å] | 27.70625(14) | 15.2751(7) |
| *c* [Å] | 30.21325(13) | 29.8809(16) |
| *α* [°] |  | 101.081(4) |
| *β* [°] | 98.4434(4) | 94.839(3) |
| *γ* [°] |  | 95.077(3) |
| vol [Å]^3^ | 13181.88(11) | 6759.4(5) |
| *Z* | 2 | 1 |
| *ρ* (calc) [g/cm^3^] | 1.221 | 1.248 |
| *μ* [mm^-1^] | 1.013 | 2.021 |
| F(000) | 5134 | 2686 |
| reflections collected | 130545 | 73852 |
| independent reflections (*R*_int_) | 46274 (0.0302) | 36612 (0.1257) |
| parameters, restraints | 3047, 693 | 3085, 345 |
| GoF on *F*^2^ | 1.037 | 1.012 |
| *R_1_* [*I* > 2*σ*(*I*)] | 0.0744 | 0.1008 |
| *wR_2_* (all data) | 0.2246 | 0.3183 |
| largest diff. peak/hole [e/Å^3^] | 0.940, -0.941 | 0.781, -0.390 |
| Flack *x* parameter | 0.208(14) | 0.098(16) |

**Supplementary Table 1.** Selected crystallographic data for the two MOFs.

**Supplementary Table 2.** Comparison of unit cell parameters of crystals structures of Na-β-CD-MOFs, previously reported and from the current work.

| **CSD Refcode** | **PULLOZ** | **ZUNVEL** | **CCDC 2456464** |
| --- | --- | --- | --- |
| ***a* (Å)** | 15.237(5) | 10.353(5) | 15.91971(8) |
| ***b* (Å)** | 10.596(5) | 19.678(5) | 27.70625(14) |
| ***c* (Å)** | 20.202(5) | 29.790(5) | 30.21325(13) |
| **α (°)** | 90 | 90 | 90 |
| **β (°)** | 108.224(5) | 90 | 98.4434(4) |
| **γ (°)** | 90 | 90 | 90 |
| **V (Å^3^)** | 3098.04 | 6069.01 | 13181.9 |
| **Spacegroup** | P2_1_ | P2_1_2_1_2_1_ | P2_1_ |
| **Reference** | ^[1]^ | ^[2]^ | **This work** |

**Supplementary Table 3.** Comparison of unit cell parameters of crystals structures of K-β-CD-MOFs, previously reported and from the current work.

| **CSD Ref- code** | **PULLUF** | **SEFWIN** | **YAMVIV** | **RIZBEL** | **HOXDOQ** | **CCDC 2456465** |
| --- | --- | --- | --- | --- | --- | --- |
| ***a* (Å)** | 15.235(5) | 15.2330(0) | 14.751(2) | 15.6904(10) | 15.2438(6) | 15.2321(5) |
| ***b* (Å)** | 10.594(5) | 10.6004(8) | 23.6860(10) | 24.6287(2) | 15.2742(6) | 15.2751(7) |
| ***c* (Å)** | 20.241(5) | 20.3014(14) | 40.870(2) | 18.8656(2) | 29.7686(13) | 29.8809(16) |
| **α (°)** | 90 | 90 | 90 | 90 | 101.314(2) | 101.081(4) |
| **β (°)** | 108.180(5) | 108.739(2) | 90 | 108.6240(10) | 94.845(2) | 94.839(3) |
| **γ (°)** | 90 | 90 | 90 | 90 | 95.071(2) | 95.077(3) |
| **V (Å^3^)** | 3103.81 | 3104.42 | 14279.7 | 6908.55 | 6733.06 | 6759.42 |
| **Space group** | P2_1_ | P2_1_ | P2_1_2_1_2_1_ | P2_1_ | P1 | P1 |
| **Reference** | ^[1]^ | ^[3]^ | ^[4]^ | ^[5]^ | ^[6]^ | **This work** |

# Infrared Spectroscopy

**
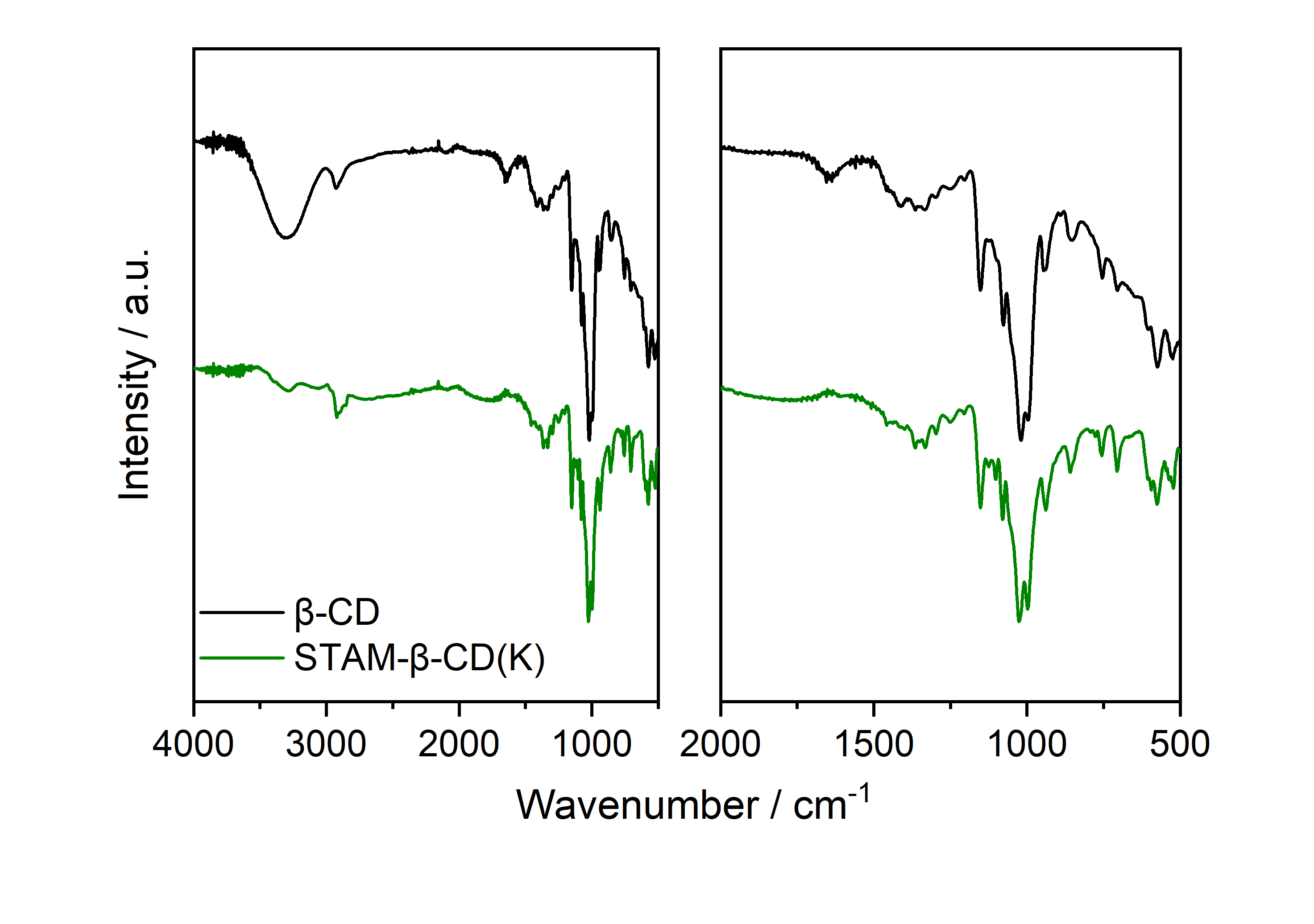
**

**Figure SI 15.** FT-IR spectra for β-CD (black) and STAM-β-CD(K) (green).

# Electron Microscopy


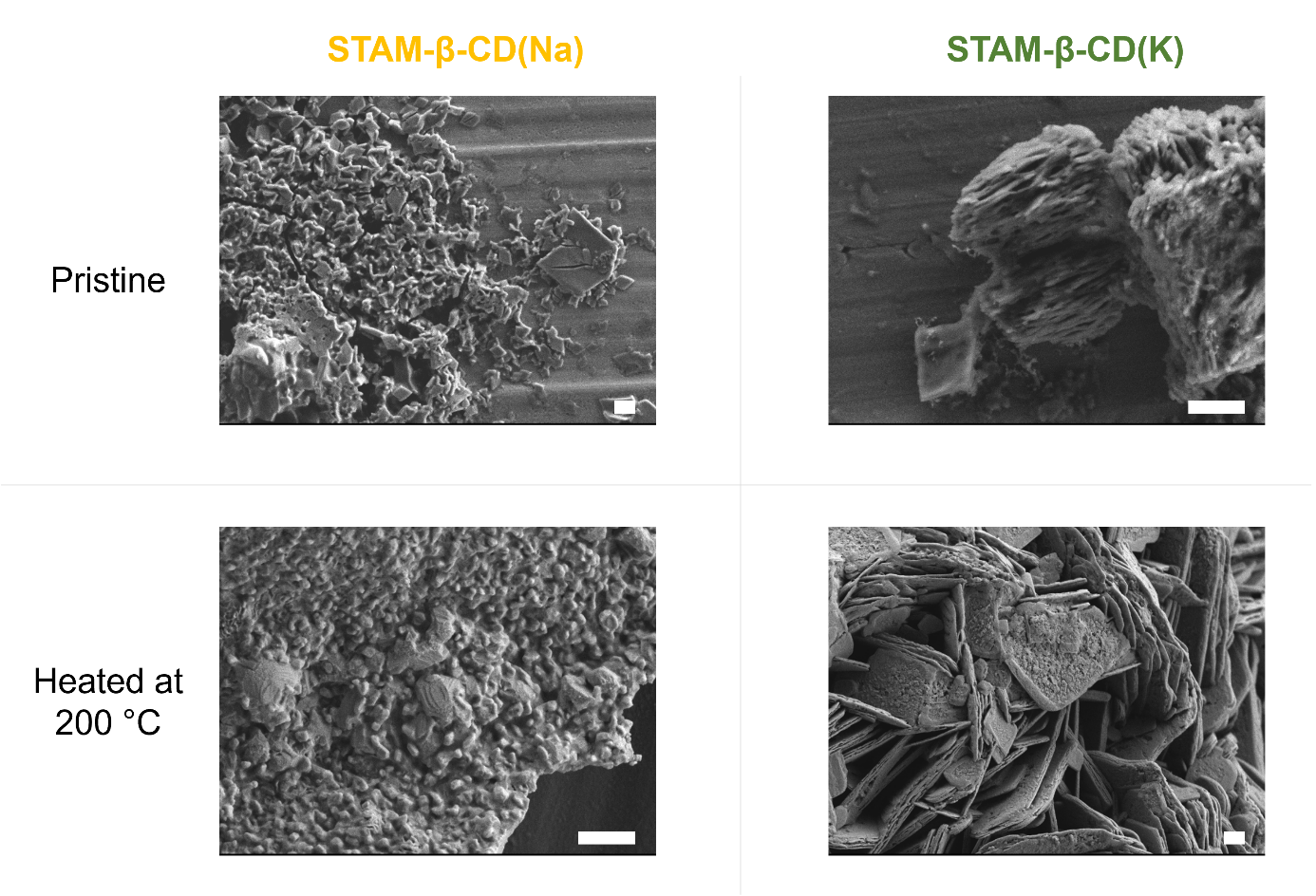


**Figure SI 16.** SEM images for samples for pristine and heated samples of STAM-β-CD(Na) and STAM-β-CD(K), scale bar: 1 μm.

# References

[1] J.-Q. Sha, L.-H. Wu, S.-X. Li, X.-N. Yang, Y. Zhang, Q.-N. Zhang, P.-P. Zhu, *J. Mol. Struct.* **2015**, *1101*, 14.

[2] H. Lu, X. Yang, S. Li, Y. Zhang, J. Sha, C. Li, J. Sun, *Inorg. Chem. Commun.* **2015**, *61*, 48.

[3] L. Huijun, L. Juan, W. Na, *J. Univ. South China (Sci. Technol.)* **2021**, *35*, 86.

[4] E. I. Koshevoi, D. G. Samsonenko, P. V. Dorovatovskii, V. A. Lazarenko, V. P. Fedin, *J. Struct. Chem.* **2021**, *62*, 577.

[5] K. Krūkle-Bērziņa, A. Mishnev, *ACS Omega* **2023**, *8*, 48221.

[6] B. A. Blight, T. I. Ahmad, H. J. Shepherd, C. S. Jennings, L. I. Ferland, S. J. Teat, J. S. Rossman, *Cryst. Growth Des.* **2020**, *20*, 43.
